# ECG signal performance de-noising assessment based on threshold tuning of dual-tree wavelet transform

**DOI:** 10.1186/s12938-017-0315-1

**Published:** 2017-02-07

**Authors:** Oussama El B’charri, Rachid Latif, Khalifa Elmansouri, Abdenbi Abenaou, Wissam Jenkal

**Affiliations:** 1 ESSI-LISTI Laboratory, National School of Applied Sciences, Ibn Zohr University, Agadir, Morocco; 2 High School of Biomedical Engineering, UM6SS University, Casablanca, Morocco

**Keywords:** ECG, De-noising, Dual tree wavelet transform, Threshold tuning, Realistic noise

## Abstract

**Background:**

Since the electrocardiogram (ECG) signal has a low frequency and a weak amplitude, it is sensitive to miscellaneous mixed noises, which may reduce the diagnostic accuracy and hinder the physician’s correct decision on patients.

**Methods:**

The dual tree wavelet transform (DT-WT) is one of the most recent enhanced versions of discrete wavelet transform. However, threshold tuning on this method for noise removal from ECG signal has not been investigated yet. In this work, we shall provide a comprehensive study on the impact of the choice of threshold algorithm, threshold value, and the appropriate wavelet decomposition level to evaluate the ECG signal de-noising performance.

**Results:**

A set of simulations is performed on both synthetic and real ECG signals to achieve the promised results. First, the synthetic ECG signal is used to observe the algorithm response. The evaluation results of synthetic ECG signal corrupted by various types of noise has showed that the modified unified threshold and wavelet hyperbolic threshold de-noising method is better in realistic and colored noises. The tuned threshold is then used on real ECG signals from the MIT-BIH database. The results has shown that the proposed method achieves higher performance than the ordinary dual tree wavelet transform into all kinds of noise removal from ECG signal.

**Conclusion:**

The simulation results indicate that the algorithm is robust for all kinds of noises with varying degrees of input noise, providing a high quality clean signal. Moreover, the algorithm is quite simple and can be used in real time ECG monitoring.

## Background

Recently, wavelet transform (WT) that localizes features in time–frequency domain has been emerged widely in ECG signal de-noising [1]. Generally, removing noises based on WT can be divided in two mainly methods. The first method is based on WT modulus maxima by holding the maximum information on the original ECG signal, which lead to a large amount of calculation [2], while the second method used by Donoho and Johnstone [3, 4] threshold the decomposed wavelet coefficients then reconstruct the signal using inverse wavelet transform. Although the efficiency of WT based thresholding method in ECG de-noising, it suffers from some shortcomings like aliasing that brings artifacts in the de-noised signal when the wavelet coefficients are processed [5]. In order to overcome those shortcomings, the dual tree wavelet transform (DT-WT) has been introduced with new properties that can enhance the reconstructed ECG signal [6]. The DT-WT was tested on ECG signal de-noising applying soft thresholding on magnitude nonlinearity [7]. However, the optimal decomposition level together with threshold value and function was not taken into consideration.

A substantial amount of studies focused their work on removing commonly known noise such as white noise. Generally, a reliable de-noising algorithm is able to remove noise from the acquired ECG signal, such as power-line interference, baseline wander, muscle noise and motion artifact and other noises with, which in different levels leads to misjudgment and deletion of standard ECG identification for the ECG feature extraction and decreases the degree of diagnostic accuracy. Moreover, with the modern tele-healthcare systems involving transmission and storage of ECG, noise also arises due to poor channel conditions. A noisy ECG may hinder the physician’s correct evaluations on patients. Therefore, removing noise from ECG signal and pre-processing has become an exclusive requirement.

On the other hand, wavelet thresholding is a viable technique for noise reduction, the value of the threshold is usually application dependent and difficult to fix in practice. Wavelet threshold function mainly includes hard thresholding and soft thresholding. The basis of these methods is quite simple, and they are easy to use in practice. Hard thresholding can retain the abrupt information in the signal, but it may generate oscillations in the reconstructed signal known as Pseudo-Gibbs phenomenon [8, 9]. Soft thresholding can further smoothen the signal than hard thresholding, and has a good continuity. However, the reconstructed signal may be distorted and has a blurred edge. Furthermore, the amplitude of the reconstructed signal will decrease significantly, in particular, the amplitude of the R wave in QRS complex will attenuate greatly, which is a crucial parameter for heart diagnosis. All these shortcomings are detrimental for cardiovascular diagnostic accuracy.

To overcome aforementioned limitations and to provide an efficient tool for the extraction of high-resolution ECG signals from recordings contaminated with background noise, the dual tree wavelet transform, which has elegant computational structure [10, 11] is investigated in this paper. The results are obtained by performing extensive simulation studies on threshold tuning. This threshold tuning is performed by varying the threshold value and function as well as the optimal decomposition level, which affects the algorithm performance on removing the noise. This performance is assessed by using a wide range of noises that are of major concern.

This paper is organized as follows; the “Methods” section is dedicated to the theoretical background on the dual tree complex wavelet transform. In this section, the materials used and the proposed algorithm are also presented. For quantitative and qualitative assessments of the algorithm performance, a set of simulations is performed in the “Results” section. These simulations are discussed and explained in the “Discussions” section. Finally, the conclusion of this study is provided in the “Conclusions” section.

## Methods

### Data acquisition

#### Synthetic ECG signal

In order to obtain free-noise ECG signal, a synthetic ECG signal is used. The dynamical model used, which is introduced by McSharry [12], can generate a realistic artificial ECG waveform. The signal is created by coupling three ordinary differential equations. The user can settle various parameters including the ECG sampling frequency, number of beats, mean heart rate and waveform morphology. In this work, the default synthetic ECG parameters were taken.

#### Colored noises

To generate these noises, the DSP System Toolbox integrated in Matlab 2014 was used. These kinds of noises are random signals that respect a well-defined shape on the frequency spectrum. Each colored noise name corresponds to the light wave frequencies of a particular color. The equation that characterizes colored noises is defined by:

1 $$S\left( f \right) \propto \frac{{\sigma_{V} }}{{\left| f \right|^{\beta } }}$$

where $S(f)$ represents the power spectral density function of a frequency $f$, $\sigma_{V}$ is the variance of the original signal; $\beta$ is the slope that distinguishes each noise color as shown in Table 1.

**Table 1:** Colored noise type based on *β* value

*β* value	Noise type
0	White
1	Pink (flicker noise)
2	Brown (Brownian motion)
−1	Blue
−2	Violet (purple noise)

#### ECG acquisition from ECG databases

To work with real ECG signal, two databases were used. The first database is the MIT-BIH Arrhythmia Database [13]. It includes 48 annotated recordings. Each record lasts about 30 min and is sampled at a frequency of 360 Hz with 11-bit resolution over a 10 mV range. The signals are extracted from two channel ambulatory ECG recordings. About 29 records are collected from a mixed population of inpatients; the remaining records are collected from outpatients. The second database used is the MIT-BIH noise stress test database [14]. This database can be classified into two classes of records. The first class includes 3 recordings of noise typical in ambulatory ECG recordings. These real noise records are baseline wander (BW), muscle artifact (MA), and electrode motion (EM) artifact. They are created using physically active volunteers and standard ECG recorders, leads, and electrodes while the second class contains 12 records that are created from two signals (118 and 119) of the MIT-BIH Arrhythmia database by adding the EM noise. All the records contained in this database are about 30 min in length having sampling frequency of 360 Hz with 12-bit resolution. We are interested in the first class since it gives us the ability to add calibrated amounts of real noise to any free-noise ECG signal. All the ECG data along with further information about these records can be collected from the two described database via [13, 14], respectively.

### Wavelet analysis

#### Wavelet transform

Since its inception, the WT has become the most powerful tool for analyzing signals in many fields of research including the analysis of non-stationary signals. Unlike the traditional Fourier transform (FT), WT provides a time–frequency analysis that can detect local, transient or intermittent components in the studied signal. It is a linear transform, which can refine a signal into multi-resolution representation using a scaled and shifted form of the mother wavelet. For practical applications, Mallat [15] has introduced a reliable and efficient algorithm to calculate the discrete wavelet transform (DWT).

Despite its success in several areas of research, the DWT suffers from several drawbacks like oscillations around singularities, shift variance and lack of directionality. Besides, the aliasing issue appears when wavelet coefficients are threshold, which causes distortion to the reconstructed signal.

#### Dual tree wavelet transform

The DT-WT was introduced by Kingsbury [6] in 1998. It is one of the most improved versions of DWT that brings new fundamental properties that overcome the limitations encountered the DWT. The DT-WT is built using two separate real filters, which represent the dual tree, to give an analytic transform. The first tree produces the real part of the complex wavelet coefficient while the second produces the imaginary part. Based on FT representation, Kingsbury has proposed to construct the DT-WT that is expressed as:

2 $$\psi \left( t \right) = \psi_{h} \left( t \right) + i \psi_{g} \left( t \right)$$

with magnitude

3 $$\left| {\psi (t)} \right| = \sqrt {\left| {\psi_{h} \left( t \right)} \right|^{2} + \left| {\psi_{g} (t)} \right|^{2} }$$

and phase

4 $$\angle \psi \left( t \right) = \arctan \left( {\frac{{\psi_{g} \left( t \right)}}{{\psi_{h} \left( t \right)}}} \right)$$

where $i = \sqrt { - 1}$, $\psi_{h} \left( t \right)$ is a real and even function, whereas $\psi_{g} (t)$ is an imaginary and an odd function. These two functions are implemented so that $\psi_{g} (t)$ is the Hilbert transform of $\psi_{h} \left( t \right)$ in order to ensure the perfect reconstruction of the decomposed signal.

### Wavelet thresholding

#### Wavelet threshold functions

**Fig. 1:**
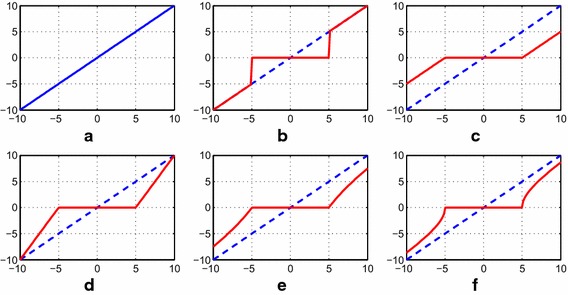
Threshold response applied to linear test signal (**a**) using the functions: **b** hard, **c** soft, **d** semi-soft, **e** non-negative garrote and **f** hyperbolic

The threshold function is used to reduce the noise in a signal by acting on the details wavelet coefficients. According to the selected threshold function, these coefficients are shrunk or scaled. There are several types of threshold estimators. We can first distinguish the hard thresholding and soft thresholding proposed by Donoho and Johnstone [3, 4]. The other threshold functions are derived from the mainly soft thresholding. In this study, the following threshold techniques are tested on the algorithm. Their performances are illustrated in Fig. 1. Their formulas are expressed as follows:

- Hard thresholding.
  5 $$R = \left\{ {\begin{array}{*{20}l} {s,} \quad {\left| s \right| \ge Th} \\ {0,} \quad {\left| s \right| Th} \\ \end{array} \begin{array}{*{20}l} { } \\ { } \\ \end{array} } \right.$$
- Soft thresholding
  6 $$R = \left\{ {\begin{array}{*{20}l} {sign(s)(\left| s \right| - Th),} & \quad {\left| s \right| \ge Th} \\ {0,} & \quad {\left| s \right| < Th} \\ \end{array} \begin{array}{*{20}l} { } \\ { } \\ \end{array} } \right.$$
- Semi-soft thresholding (S–S)
  7 $$R = \left\{ {\begin{array}{*{20}l} { } \\ {\begin{array}{*{20}l} {s,} & \quad {\left| s \right| < Th_{2} } \\ {sign(s)\frac{{\left( {s - Th_{1} } \right)*Th_{2} }}{{Th_{2} - Th_{1} }},} & \quad {Th_{2} < \left| s \right| \le Th_{1} } \\ {0,} & \quad {\left| s \right| < Th_{1} } \\ \end{array} } \\ { } \\ \end{array} } \right.$$
- Non-negative Garrote thresholding (N-NG)
  8 $$R = \left\{ {\begin{array}{*{20}l} {sign(s)\left( {\left| s \right| - \frac{{Th^{2} }}{\left| s \right|}} \right),} & \quad {\left| s \right| \ge Th} \\ {0,} & \quad {\left| s \right| < Th} \\ \end{array} } \right.$$
- Hyperbolic thresholding (HYP)
  9 $$R = \left\{ {\begin{array}{*{20}l} {sign(s)\sqrt {s^{2} - Th^{2} } ,} & \quad {\left| s \right| \ge Th} \\ {0,} & \quad {\left| s \right| < Th} \\ \end{array} } \right.$$

In these techniques, the variable $R$ refers to the resulting signal from threshold function, $s$ represents the wavelet coefficients and $Th$ is the threshold value. In the case of semi-soft thresholding, this function introduces two threshold values $Th_{1}$ and $Th_{2}$, where $Th_{1} < Th_{2}$.

#### Threshold value selection

Among the critical parameters, that affect the quality of noise suppression, is the threshold value. According to the selected value, the de-noised ECG signal could either retain some interferences or have some distortion and discontinuities, depending on whether the threshold value was too small or overly large value. The common threshold values used in the literature [16, 17] are defined as follows:

- Universal threshold
  10 $$Th_{1} = \sigma \sqrt {2\log N}$$
- Universal threshold level dependent
  11 $$Th_{2} = \sigma_{j} \sqrt {2\log n_{j} }$$
- Universal modified threshold level dependent
  12 $$Th_{3} = \sigma_{j} \frac{{\sqrt {2\log n_{j} } }}{{\sqrt {n_{j} } }}$$
- Exponential threshold
  13 $$Th_{4} = 2^{{\left( {\frac{j - J}{2}} \right)}} \sigma_{j} \sqrt {2\log N}$$
- Exponential threshold level dependent
  14 $$Th_{5} = 2^{{\left( {\frac{j - J}{2}} \right)}} \sigma_{j} \sqrt {2\log n_{j} }$$
- Minimax threshold
  15 $$Th_{6} = 0.3936 + 0.1829 \times \left( {\frac{{\log n_{j} }}{\log 2}} \right)$$
- The modified unified threshold [17]
  16 $$Th_{7} = \sigma_{j} \frac{{\sqrt {2\log (N)} }}{\log (j + 1)}$$

where $N$ denotes the length of the original signal, $n_{j}$ represents the length of the signal at $j$-th scale, while $\sigma_{j}$ is the standard deviation on $j$-th decomposition level that is expressed as:

17 $$\sigma_{j} = \frac{{MAD(\left| {d_{j} } \right|)}}{0.6745}$$

Here, $MAD$ is the median absolute deviation and is defined as:

18 $$MAD = median\left( {\left| {d_{j} - median\left( {d_{j} } \right)} \right|} \right)$$

The value of $\sigma$ is calculated from each level detail coefficients except the universal threshold case, which is calculated from the first level detail coefficients.

### The proposed algorithm

**Fig. 2:**
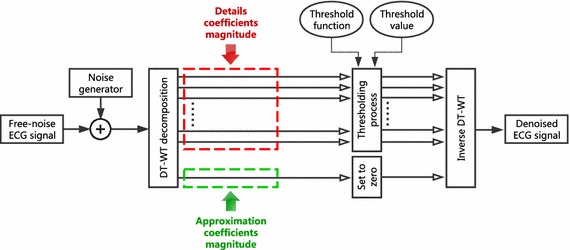
Flowchart of the proposed DT-WT de-noising algorithm

Since we want to implement this algorithm in an embedded system in our future work, the noise reduction procedure established in this study is quite simple. This method benefits the power of time–frequency analysis that offers the DT-WT while using a minimum amount of computation. The proposed ECG signal de-noising algorithm using DT-WT is illustrated in Fig. 2 of which different steps are explained as follows:

#### Free-noise ECG signal

To have a clean real ECG signal seems to be difficult. In our study, we first use the synthetic ECG signal for visual performance evaluation of noise reduction algorithm. These signals can be assumed as nearly free-noise signals. Afterward, we process the other signals of the described databases.

#### Noise generator

To generate noise, we create a function in Matlab that can generate various types of noises, including white noise, colored noise (flicker, Brownian noise, blue, and purple), baseline wander noise (BW), electromyogram noise (EM), and motion artifact (MA). These interferences are inserted into a clean ECG signal with a desired value of signal-to-noise-ratio.

In addition, the function can also generate a combined noise (CN). This CN reflects the realistic noise case that prevents an ECG recording. It is a composition of EM, MA, and BW noise. Each of the three noises can be controlled as described in the following expression:

19 $${\text{CN}} = \frac{{wbw \times {\text{BW}} + wem \times {\text{EM}} + wma \times {\text{MA}}}}{wbw + wem + wma}$$

where $wbw$, $wem$, and $wma$ are the weights of baseline wander noise, electromyogram noise, and motion artifact, respectively. Each weight defines the added noise percentage. For example, if we choose $wbw = 2$, $wem = 2$, and $wma = 5$, means that motion artifact is the predominant noise in the noisy ECG signal.

#### DT-WT ECG decomposition level

**Fig. 3:**
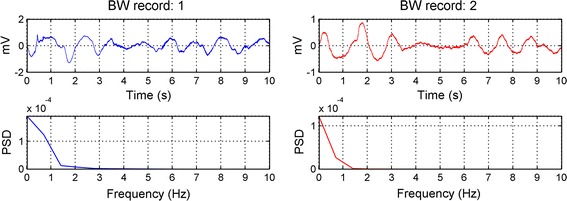
The corresponding power spectrum of the two ‘bw’ records of length 10 s from the noise stress test database

The decomposition level depends on the baseline wandering frequency, since it is the lowest noise frequency among other noises. To estimate the frequency of baseline wander, the two ‘bw’ records of [14] are taken into consideration. The power spectrum of each record is calculated. From Fig. 3, we can observe that the frequency range of both records is approximately concentrated below $f_{BL} = 1 \;{\text{Hz}}$. To select the appropriate decomposition level $J$, we used the formula described in [16]:

20 $$J = ceil\left( {\log_{2} \left( {\frac{{F_{max} }}{{f_{BL} }}} \right)} \right)$$

where the $ceil(x)$ function rounds the element $x$ to the nearest integer greater than or equal to that $x$, $F_{max}$ represents the highest frequency components that respect the Nyquist Theorem, whereas, $f_{BL}$, which is equal to 1, is the baseline wander frequency. Since each database has its sampling frequency, $F_{max}$ will change its value from database to another.

#### Zeroing approximation coefficients

Since the decomposition level is determined, the approximation coefficients magnitude at level $J$ are set to zero to suppress the baseline wander noise.

#### Details magnitude threshold

The details coefficients representing the high frequency of the signal are quantified up to a level $X$. This level $X$ is determined empirically through a set of simulations. To select the optimal values, all threshold values and functions presented in this work have been tested on the algorithm.

#### Reconstruct ECG signal

The processed details coefficients at each level, all together with the vanished approximation coefficients at level $J$ are inversely transformed using the inverse dual tree complex wavelet transform to get the clean ECG signal.

### Evaluation parameters

To quantify the algorithm performance and compare it with other methods, we took the most significant and widespread parameters in literature. In the following expressions, $x_{n} \left( n \right)$ means the studied original signal $x_{0} \left( n \right)$ corrupted by noise, while $x_{r} \left( n \right)$ represents the de-noised signal that is reconstructed from the algorithm.

- Mean square error

21 $$MSE = \frac{1}{N}\mathop \sum \limits_{n = 0}^{N - 1} \left[ {x_{0} \left( n \right) - x_{r} \left( n \right)} \right]^{2}$$

- Signal-to-noise-ratio

This parameter measures the amount of noise in a signal. This noise is evaluated at two levels, the noisy input signal in the case of $SNR_{in}$ and the output reconstructed signal for $SNR_{out}$.

22 $$SNR_{in} \left( {\text{dB}} \right) = 10\log_{10} \left( {\frac{{\mathop \sum \nolimits_{n = 0}^{N - 1} \left[ {x_{0} \left( n \right)} \right]^{2} }}{{\mathop \sum \nolimits_{n = 0}^{N - 1} \left[ {x_{0} \left( n \right) - x_{n} \left( n \right)} \right]^{2} }}} \right)$$

23 $$SNR_{out} \left( {\text{dB}} \right) = 10\log_{10} \left( {\frac{{\mathop \sum \nolimits_{n = 0}^{N - 1} \left[ {x_{0} \left( n \right)} \right]^{2} }}{{\mathop \sum \nolimits_{n = 0}^{N - 1} \left[ {x_{0} \left( n \right) - x_{r} \left( n \right)} \right]^{2} }}} \right)$$

The overall signal-to-noise-ratio is assessed using the $SNR$ improvement.

24 $$SNR_{imp} \left( {\text{dB}} \right) = SNR_{out} \left( {\text{dB}} \right) - SNR_{in} \left( {\text{dB}} \right)$$

From Eqs. (24) and (21), the greater the $SNR_{imp}$ is, the better de-noising performance is achieved. Conversely, as the $MSE$ parameter is small as the distortion is low in the reconstructed signal.

## Results

A set of analyses is performed to achieve the promised results. For simplicity and practical implementation reasons, the studied signal is decomposed using ‘farras’ filter [18].

### Baseline wander removal

**Fig. 4:**
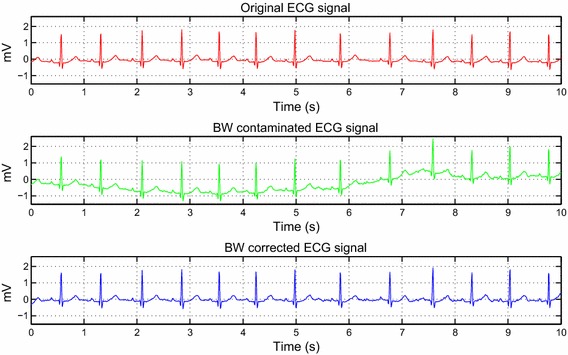
Baseline wander noise corrected ECG signal

To validate the algorithm performance at the removal of baseline wander, we corrupt the synthetic ECG signal by ‘bw’ noise from the noise generator using −5 dB $SNR_{in}$. The added noise value is selected in such a way that it can be seen in Fig. 4.

### Threshold tuning

Fixing the random numbers at white noise generation, the threshold values and functions presented in this paper are all tested and the $SNR_{imp}$ is then calculated as shown in Table 2.

**Table 2:** The *SNR* improvement for various threshold values and functions

Threshold function	*SNR*_*in*_ (dB)	*Th*_*1*_	*Th*_*2*_	*Th*_*3*_	*Th*_*4*_	*Th*_*5*_	*Th*_*6*_	*Th*_*7*_
Soft	−5	3.20389	8.98377	0.25384	1.62297	1.47873	8.25054	9.91075
	0	3.20123	8.53072	0.25589	1.61892	1.47919	4.49104	8.72283
	5	3.19821	7.74215	0.26424	1.63262	1.50036	−0.02684	6.82608
S–S	−5	1.68416	3.31768	0.00031	0.02849	0.01377	8.51847	11.02541
	0	1.67689	3.33545	0.00033	0.03438	0.01846	4.55702	10.40910
	5	1.66978	3.50081	0.00042	0.05118	0.02850	−0.02684	9.22058
HYP	−5	2.35838	5.39335	0.00599	0.23616	0.18567	8.49517	10.96391
	0	2.35281	5.34901	0.00627	0.24495	0.19283	4.56183	10.45635
	5	2.34758	5.38295	0.00713	0.27530	0.21732	−0.02684	9.39921
N-NG	−5	2.80969	7.16647	0.01163	0.42158	0.34169	8.31101	10.62271
	0	2.80490	7.04860	0.01217	0.43125	0.35085	4.50462	10.16068
	5	2.80054	6.89711	0.01375	0.47023	0.38413	−0.02684	9.03560
Hard	−5	1.42053	2.61142	0.00031	0.01829	0.00901	8.87432	10.86859
	0	1.41609	2.60485	0.00029	0.02486	0.01048	4.67213	10.25426
	5	1.40484	2.77515	0.00042	0.03542	0.02116	−0.02684	9.32698

**Fig. 5:**
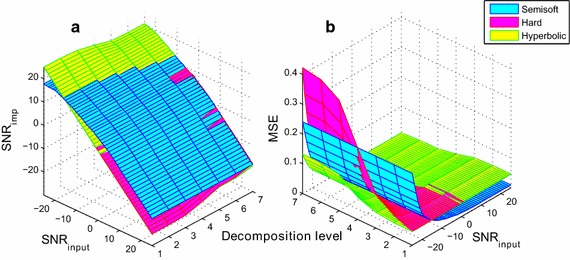
Threshold tuning based on **a** $SNR$ improvement and **b** MSE

In order to choose the suitable threshold function as well as the decomposition level, the $SNR_{in}$ and the decomposition level are ranged from −25 to 25 dB and from 1 to *J* level, respectively. The simulation is then performed under the white noise using the three favorable threshold functions that are semi-soft, hard and hyperbolic. By varying all these parameters, the $SNR_{imp}$ and $MSE$ are represented in a surface as illustrated in Fig. 5a and b, respectively.

### De-noising results based on the optimized threshold

#### De-noising results using the synthetic ECG signal

**Fig. 6:**
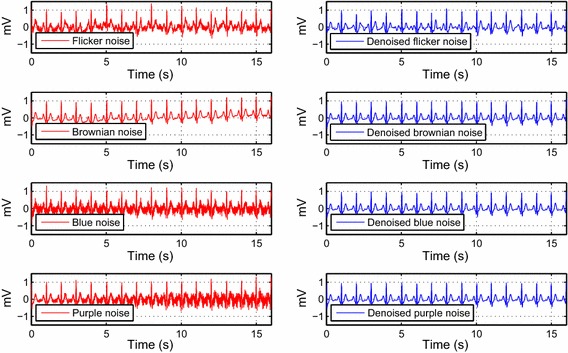
Colored noise removal from the ECG signal using the tuned parameters on DT-WT

To assess the algorithm performance, the tuned parameters are used on the synthetic ECG signal using colored noises with input $SNR$ 5 dB as illustrated in Fig. 6.

**Fig. 7:**
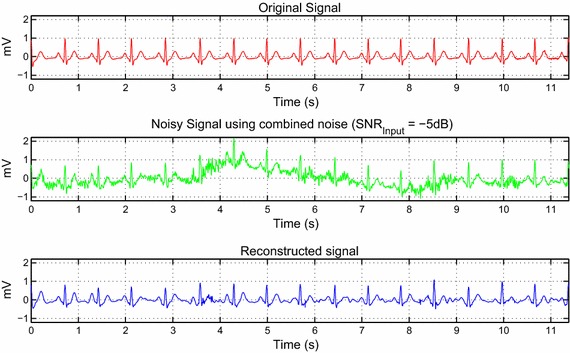
Real noise reduction of an ECG signal using tuned parameters on DT-WT

During ECG signal acquisition, we encountered an ECG signal contaminated by a realistic noise that includes baseline wander, motion artifacts and electromyogram noise. Hence, from Eq. 19, this realistic noise is applied to a clean ECG signal using input $SNR$ −5 dB as illustrated in Fig. 7. The weights used are 5, 10, and 10 for $wbw$, $wem$, and $wma$, respectively.

For quantitative evaluation, the $SNR$ improvement is computed for each type of noise applied to the synthetic ECG signal with input $SNR$ 5 dB using the hyperbolic threshold function. Table 3 shows the results based on $SNR$ improvement and $MSE$.

**Table 3:** ECG de-noising performance for various types of noise

ECG signal corrupted by 5 dB *SNR*_*in*_	*SNR*_*imp*_	*MSE*
Flicker noise	5.62309	0.00399
Brownian noise	14.83924	0.00048
Blue noise	13.19360	0.00070
Purple noise	14.65982	0.00050
BW noise	15.24564	0.00044
EM noise	5.65466	0.00397
MA noise	7.21953	0.00277
Combined noise	10.85304	0.00120

#### De-noising results using MIT-BIH signals

**Fig. 8:**
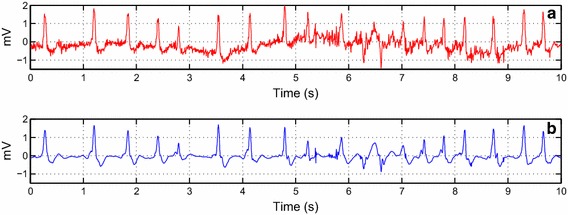
The de-noising result of the record no. 203 collected from the MIT-BIH Arrhythmia. **a** The original record. **b** The de-noised record

**Fig. 9:**
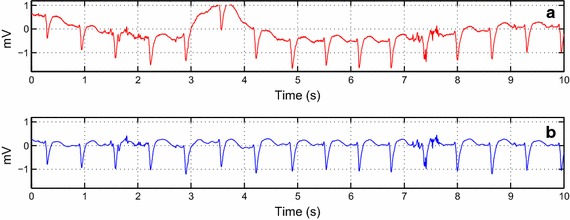
The de-noising result of the record no. 109, lead V1 collected from the MIT-BIH Arrhythmia. **a** The original signal. **b** The de-noised signal

**Fig. 10:**
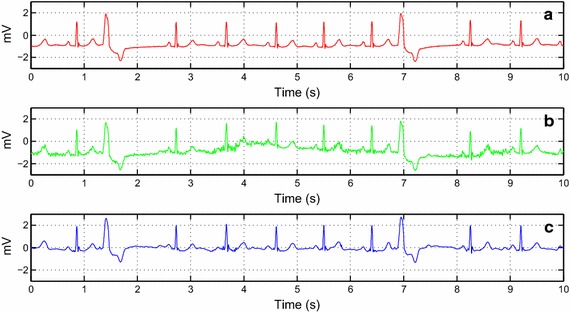
The de-noising result of the record no. 119 collected from the MIT-BIH Arrhythmia database. **a** The original ECG record noise free. **b** The contaminated ECG record with input $SNR$ 10 dB using weights 10, 10 and 15 for $wbw$, $wem$ and $wma$. **c** The de-noised record

**Fig. 11:**
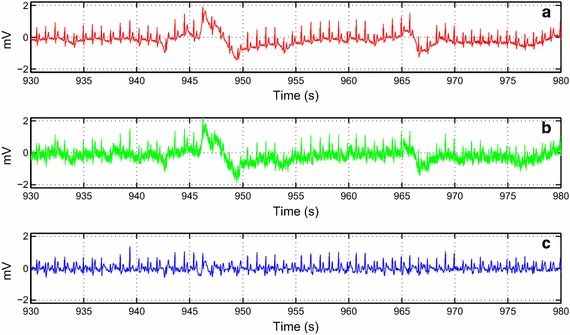
The de-noising result of the record no. 111 collected from the MIT-BIH Arrhythmia. **a** The original noisy ECG record. **b** The contaminated ECG record with 5 dB flicker noise. **c** The de-noised record

**Fig. 12:**
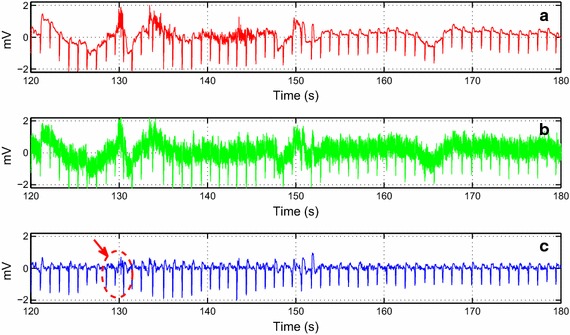
The de-noising result of the record no. 108, lead V1 collected from the MIT-BIH Arrhythmia. **a** The original noisy ECG record. **b** The contaminated ECG record with 5 dB white noise. **c** The de-noised record

To materialize the algorithm performance, we took some ECG signals from the MIT-BIH Arrhythmia database. The de-noising algorithm is then applied to signals 203, 109, 119, 111 and 108 as shown in Figs. 8, 9, 10, 11 and 12, respectively.

## Discussions

The simulation results are established in three separate steps. Before we discuss each subsection result, it should be noted here that to exhibit the algorithm performance and to provide a visual noisy ECG signal on all the figures, the input $SNR$ noise is added with values representing a strong background noise. In the first subsection, we can distinguish the result of baseline wander de-noising since it is independent of threshold process of the detail coefficients. The BW noise used here is a real noise and is taken from noise stress database [14]. From Fig. 4, it is obvious that BW noise is perfectly removed without introducing distortion to the original ECG signal.

To choose the optimal threshold, all the previously described threshold functions and values are tested in the threshold tuning subsection. According to Table 2, the modified unified threshold value $Th_{7}$ gives the best result over all other threshold values. It can effectively reduce all kinds of high frequency and low frequency noises while preserving the amplitude and characteristics of ECG signals. By against, the threshold functions exhibit low variation in *SNR* improvement. It may be noted that the hard, soft and semi-soft functions slightly surpasses the two other functions. Once these threshold parameters are settled, we vary both the decomposition level and the input noise to observe the suitable threshold level. We can see that the amount of added noise and the decomposition level greatly influence the choice of the threshold function. By analyzing the added noise percentage in Fig. 5a and b, the semi-soft function is most suitable for positive values of $SNR_{in}$, while the hyperbolic function is most adapted when $SNR_{in}$ is negative. For the remainder of the simulations, the hyperbolic function is opted since it provides a low distortion in the entire range of $SNR_{in}$. On the other hand, the threshold decomposition level is set empirically by selecting the level that affords a small $MSE$ value regardless the background power noise. From Fig. 5b, we can see that the optimal decomposition level is 3. Since the signal is decomposed to a level based on its sampling frequency, the coefficients of the details magnitude are threshold up to level $(J - 4)$. This means that the threshold level will change according to the sampling frequency of the original signal.

The last subsection of results brings together all the set parameters to evaluate the algorithm performance. This evaluation is performed initially on the synthetic ECG signal. From Fig. 6, we can clearly see the efficiency of this method on colored noises. In the case of flicker noise, we can notice a minor distortion in the de-noised signal. However, the useful information of the signal remains intact. The combined noise that represents the real noise is also tested on the synthetic ECG signal, as seen in Fig. 7. The input noise value, as well as the weights, were chosen to have a visual strong background noise. Although the ECG signal has strong background noise, the reconstructed signal preserves the QRS complex. The P and T waves have been slightly distorted in some parts of the signal.

To summarize noise removal from the synthetic ECG signal, we calculated the $SNR$ improvement and $MSE$ for all kinds of noises using 5 dB $SNR$ input. For comparison purposes, this value of input noise is chosen as the one used in a recently published work [19]. From Table 3, we can observe that flicker, EM, and MA noises are difficult to remove from ECG signal. This inconvenience is remarkable through $SNR$ improvement values.

The assessment is also expanded to signals from the MIT-BIH Database. In Figs. 8 and 9, we worked with two noisy ECG signals. We can visually observe the effectiveness of the algorithm even when changing the lead as illustrated in Fig. 9. In Fig. 10, we chose the record no. 119 from MIT-BIH Arrhythmia database that has some ectopic beats. We corrupted this record by a real noise. We can note that these ectopic beats are preserved in the reconstructed signal. Figure 11 shows the de-noising process of the record no 111 (Fig. 11a). We corrupted this noisy ECG signal by flicker noise (Fig. 11b), which is an electronic noise that is always present in some passive components like the resistors in the ECG recorder. We can observe that the algorithm can effectively remove this mixture of noises (Fig. 11c). In Fig. 12a, we took another noisy ECG signal from the MIT-BIH database. We apply the conventional white noise to this record as illustrated in Fig. 12b. We can clearly see the robustness of the algorithm on the white noise de-noising. We can notice a minor distortion in the de-noised signal (Fig. 12c). However, the clinical parameters like R peaks can be easily detected in the de-noised signal.

To compare the algorithm performance with the conventional WT and DT-WT based ECG de-noising methods, the proposed method was tested on the MIT-BIH arrhythmia database. The result of $SNR$ formula used in [7] is computed on our proposed method and is listed in Table 4. According to the results and the biomedical specialists, it is obvious that our proposed algorithm can achieve higher performance than the stated methods.

**Table 4:** Comparison of the conventional WT and DT-WT based methods with the proposed DT-WT for MIT-BIH arrhythmia records

Record no.	*SNR*		
	DWT	DT-WT	Proposed
100	42.6534	45.8309	98.5023
101	48.4172	49.3955	100.1488
102	45.4918	49.8727	100.8777
103	52.2014	55.4198	106.6181
104	54.1384	57.5161	105.5597
105	62.8240	66.1887	97.0638
106	47.4558	51.8933	93.6908
107	54.5761	55.5218	105.0257
108	43.3222	48.1617	90.3493
109	51.2570	53.7656	113.8190
111	33.9796	37.2333	96.6799

## Conclusions

In this study, threshold tuning of dual tree wavelet transform was applied to reduce noise in ECG signals. The initial simulations were conducted on synthetic ECG signal and were extended to MIT-BIH arrhythmia database. Threshold tuning was performed empirically based on the optimal threshold function, the optimal threshold value, and the suitable decomposition level. The study was extended to realistic and colored noises. The effectiveness of the proposed method was assessed through quantitative evaluation and visual inspection using a set of simulations from the standard database. The proposed technique achieves outstanding results over ordinary DT-WT based ECG de-noising methods in the presence of all kinds of noises. Furthermore, the proposed algorithm is simple to embed in real time application and is able to be investigated in QRS identification as well, which is the purpose of our future work

## Abbreviations

BW: baseline wander; CN: combined noise; DWT: discrete wavelet transform; DSP: digital signal processing; DT-WT: dual tree wavelet transform; ECG: electrocardiogram; EM: electromyogram; FT: Fourier transform; HYP: hyperbolic; Ma: motion artifact; MSE: mean square error; N-NG: non-negative garrote; S-S: semi-soft; WT: wavelet transform.

### List of symbols

$\beta {:}$ the slope of the power spectral density of colored noise; $\propto{:}$ represents the direct proportionality between two variables; $\sigma {:}$ the standard deviation of the first detail coefficients level; $\sigma_{j} {:}$ the standard deviation of the detail coefficients at j level; $\sigma_{v} {:}$ the variance of a given signal; $\psi (t) {:}$ the analytic dual tree wavelet function; $\psi_{h} (t) {:}$ the real part of the dual tree wavelet function; $\psi_{g} (t){:}$ the imaginary part of the dual tree wavelet function; $\left| {\psi (t)} \right| {:}$ the magnitude (or modulus) of the dual tree wavelet function; $\angle \psi \left( t \right) {:}$ the argument (or phase) of the dual tree wavelet function; $ciel {:}$ rounds a real number to the nearest integer greater than or equal to that number; $d_{j} {:}$ the detail wavelet coefficients of the j-th decomposition level; $f {:}$ the frequency of a given signal; $f_{BL} {:}$ the baseline wander frequency; $F_{max} {:}$ the Nyquist frequency (sampling frequency); $j {:}$ the j-th level (or scale) of the dual tree wavelet coefficients; $J {:}$ the last decomposition level of the dual tree wavelet transform; $MAD {:}$ the median absolute deviation of a given signal; $median {:}$ the median value of a given signal; $MSE {:}$ the mean square error of an estimator; $N {:}$ the length of the original signal; $n_{j} {:}$ the length of the j-th level of the dual tree wavelet coefficients; $R {:}$ the reconstructed signal from a threshold function; $s {:}$ the wavelet coefficients of a given signal; $sign {:}$ an odd function that extracts the sign of the wavelet coefficients; $SNR_{imp} {:}$ the signal-to-noise-ratio improvement; $SNR_{in} {:}$ the input signal-to-noise-ratio; $SNR_{out} {:}$ the output signal-to-noise-ratio; $Th {:}$ the threshold value; $wbw {:}$ the weight of baseline wander noise; $wem {:}$ the weight of electromyogram noise; $wma {:}$ the weight of motion artifact noise;; $X{:}$ the threshold level of the algorithm (is defined empirically from Fig. 5); $x_{n} \left( n \right) {:}$ the noisy ECG signal; $x_{0} \left( n \right) {:}$ the original ECG signal; $x_{r} \left( n \right) {:}$ the de-noised EC signal.
